# HIT-Cas9: A CRISPR/Cas9 Genome-Editing Device under Tight and Effective Drug Control

**DOI:** 10.1016/j.omtn.2018.08.022

**Published:** 2018-09-01

**Authors:** Chen Zhao, Yingze Zhao, Jingfang Zhang, Jia Lu, Li Chen, Yue Zhang, Yue Ying, Junjun Xu, Shixian Wei, Yu Wang

**Affiliations:** 1 State Key Laboratory of Stem Cell and Reproductive Biology, Institute of Zoology, Chinese Academy of Sciences, Beijing 100101, China; 2 University of Chinese Academy of Sciences, Beijing 100049, China; 3 Institute for Stem Cell and Regeneration, Chinese Academy of Sciences, Beijing 100101, China; 4 College of Biological Sciences, China Agricultural University, Beijing 100193, China

**Keywords:** CRISPR/Cas9, genome editing, drug inducible, stem cells

## Abstract

The CRISPR/Cas9 enabled efficient gene editing in an easy and programmable manner. Controlling its activity in greater precision is desired for biomedical research and potential therapeutic translation. Here, we engrafted the CRISPR/Cas9 system with a mutated human estrogen receptor (ER^T2^), which renders it 4-hydroxytamoxifen (4-OHT) inducible for the access of genome, and a nuclear export signal (NES), which lowers the background activity. Tight and efficient drug-inducible genome editing was achieved across several human cell types, including embryonic stem cells (ESCs) and mesenchymal stem cells (MSCs), upon vigorous optimization. Optimized terminal device, which we named hybrid drug inducible CRISPR/Cas9 technology (HIT-Cas9), delivered advantageous performances over several existing designs. Such architecture was also successfully applied to an orthogonal Cas9. The HIT-Cas9 system developed in this study will find broad utility in controlled editing of potentially any genomic loci.

## Graphical Abstract

**Figure:**
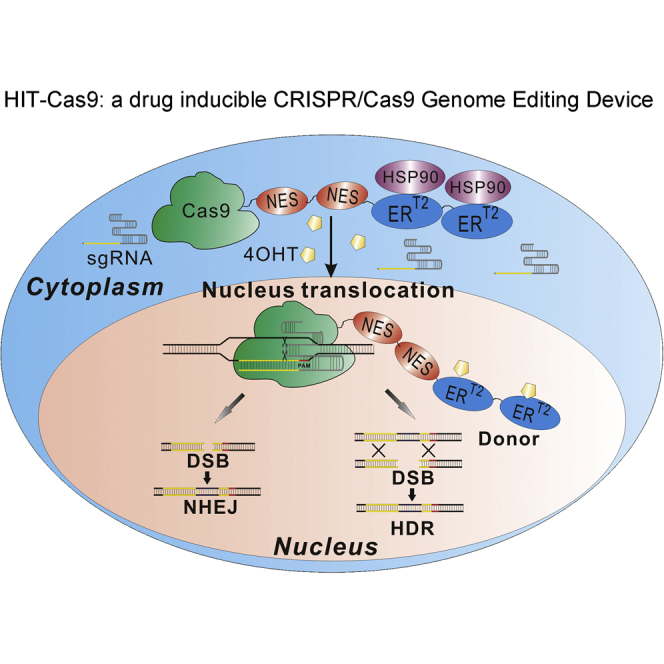

## Introduction

In the CRISPR/Cas9 system, guide RNA (gRNA) directs Cas9 protein to its complementary DNA adjacent to a protospacer-adjacent motif (PAM), a species specific requirement for Cas9 recognition.^1^, ^2^, ^3^ gRNA complementation and PAM requirement together confer selectivity for target DNA.^1^ Upon binding, DNA cleavage mediated by the Cas9-gRNA machinery leads to either error prone non-homologous end joining (NHEJ) or precise homology-directed repair (HDR).^1^, ^2^, ^3^ Further, the CRISPR/Cas9 system has been repurposed for other molecular functions, including transcription activation, repression, genomic DNA labeling, and epigenetic programming, by coupling with various effectors.^4^, ^5^

Drug control of genetic events allows dissection of gene functions in greater precision, which also reduces undesired off-target events, thus providing potential avenues toward safer gene therapies. One of the most widely used drug-inducible systems in genetic research coupled Cre recombinase with estrogen receptor (ER).^6^, ^7^ ER localizes to the cytoplasm and traffics to the nucleus upon ligand binding. Consequently, upon fusion with an ER domain, the access of Cre to the loxP sites in the engineered genome is under control of an ER ligand.^8^ ER^T2^, which contains three mutations, G400V, M543A, and L544A, selectively responds to a synthetic ligand 4-hydroxytamoxifen (4-OHT) over β-estradiol, the endogenous ER ligand, a property crucial for tight drug control.^9^

Encouraged by the wide utility of CreER^T2^, it is tempting to harnessing the power of the CRISPR/Cas9 with drug control by coupling it with ER^T2^. Here, we engineered and optimized such a system, which we named hybrid inducible CRISPR/Cas9 technology (HIT-Cas9). Tight and efficient genome editing was accomplished in a 4-OHT-inducible fashion across multiple human cell types, including ESCs and MSCs with clinical utilities, through combinatory engineering of Cas9, ER^T2^, and nuclear export signal (NES). In head-to-head comparisons, our terminal HIT-Cas9 system delivered better performances than several designs published previously, including an ER^T2^-based iCas system reported lately, while this study was in progress.^10^, ^11^, ^12^, ^13^, ^14^, ^15^ We also demonstrated that, in addition to its tightness and efficiency, drug induction of HIT-Cas9 is rapid, tunable, selective to synthetic 4-OHT over the endogenous β-estradiol, and without obvious off-target activities. Further, the terminal optimized architecture developed in this study can be adopted directly to an orthogonal Cas9 species. Consequently, the HIT-Cas9 system developed herein provides a powerful tool for effective drug-inducible editing of potentially any genomic loci.

## Results

### Design and Optimization of Cas9 ER^T2^ Fusion Constructs for Drug-Inducible Genome Editing

**Figure 1:**
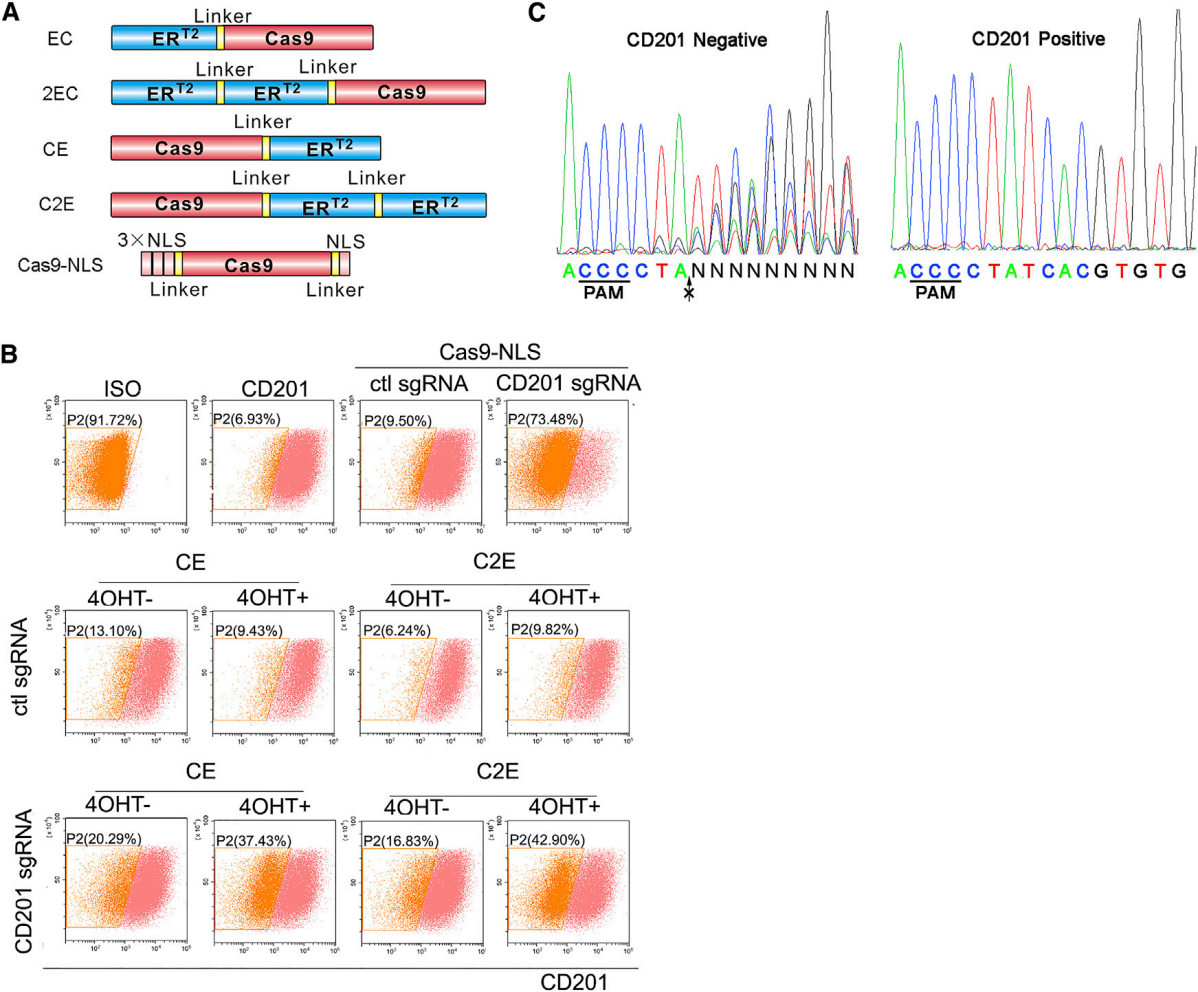
Design and Characterization of ER^T2^ Fused Cas9 Constructs (A) Schematics (not drawn to scale) of various fusion constructs of Cas9 and ER^T2^. Cas9-NLS was used as positive control (PC). (B) NHEJ induced CD201 knockout of CE and C2E examined using flow cytometry. Representative plots showed the percentage of CD201-negative cells with or without the induction of 4-OHT. ISO, 293T cells incubated with antibody isotype control; CD201, 293T cells incubated with anti-CD201 antibody conjugated with PE-Vio770 fluorophore. (C) Sanger sequencing results demonstrating NHEJ events using control Cas9-NLS plasmid. CD201-negative and -positive cell populations were sorted by flow cytometry. The arrow denotes cleavage site. Data showed mean ± SD. n = 3 biological replicates.

We first envisioned to subject Cas9 to 4-OHT regulation via fusion with ER^T2^ (Figure 1A). According to a previous study, fusion of two ER^T2^ domains to Cre appeared to exert additive effect for lowering background activity.^16^ Therefore, we fused one or two ER^T2^ domains to either the N or C terminus of Cas9 (Figure 1A). Meanwhile we identified multiple single chimeric guide RNAs (sgRNAs) showing different potency in a luciferase reporter assay, in which DNA cleavage restores a functional luciferase reading frame via single strand annealing (SSA) (Figures S1A, S1B, and S1D; Table S1).^17^ Using these sgRNAs, we found that ER^T2^ fusion to the C terminus of Cas9 (CE and C2E) less compromised its nuclease activity (Figures S1C and S1E–S1G), consistent with a previous finding that the C-terminal domain of Cas9 is more suitable than its N terminus for functional domain insertions.^18^ We also observed that tandem fusion of two ER^T2^ domains consistently maintained Cas9 activity at a level comparable to one ER^T2^ fusion construct (Figures S1C and S1E–S1G). Given that ER^T2^-based drug control is based on its nuclear translocation, we introduced EGFP to the N terminus of Cas9 in these four Cas9-ER^T2^ fusion hybrids and examined their subcellular distribution (Figure S2A). The results showed that 4-OHT only induced nucleus accumulation of the EGFP::Cas9 constructs with C-terminal ER^T2^ fusions (Figures S2B and S2C), consistent with our observations in the nuclease activity assays (Figures S1C and S1E–S1G).

We thus focused on C-terminal fusion constructs and examined their drug-inducible performances in endogenous genome editing. To this end, we used a sgRNA that effectively targets the 5′ coding region of CD201 (also known as PROCR), a cell-surface protein highly expressed in HEK293T cells, and examined NHEJ-induced gene knockout using flow cytometry (Figure 1B; Table S1). A population shift to CD201-negative range using a control Cas9 construct tagged with a nucleus-localization signal peptides (NLS) indicates successful knockout (Figure 1B). NHEJ events were found enriched in CD201-negative cell population upon Sanger sequencing (Figure 1C). As a result, Cas9 fused with two ER^T2^s (C2E) introduced lower background in the absence of 4-OHT in comparison with that fused with one ER^T2^ (CE) (Figure 1B), consistent with a previous report of additive effect of two ER^T2^ domains fused with Cre for lower background activity.^16^ Therefore, we focused on the C2E design in the following experiments.

To examine both NHEJ and HDR events induced by C2E, we next employed a traffic-light reporter (TLR) assay (Figure S3A).^19^ We used a cell line with the reporter stably integrated in the genome; thus, the drug-inducible C2E activity based on its nucleus translocation can be resolved. In this reporter construct, the target sequence of the hOct-4 (also known as POU5F1) sgRNA3, the most potent among the three being tested (Table S1; Figure S1D), was inserted in the GFP coding region. Both GFP and mCherry are out of frame. A proportion of insertions and deletions (indels) mediated by NHEJ events at the target sequence result in frameshifts that restore the mCherry reading frame. HDR with a donor GFP fragment leads to reconstitution of the GFP reading frame. Consequently, mCherry and GFP fluorescence probes NHEJ and HDR events, respectively. Using the TLR assay, we found that both NHEJ and HDR events mediated by the C2E construct were markedly induced by 4-OHT (Figures S3B and S3C). However, in both TLR assays (Figures S3B and S3C) and previous CD201 knockout assay (Figure 1B), significant background activities in the absence of 4-OHT were observed, thus warranting further optimization.

### Introduction of NES Peptides to Lower Background Activity

**Figure 2:**
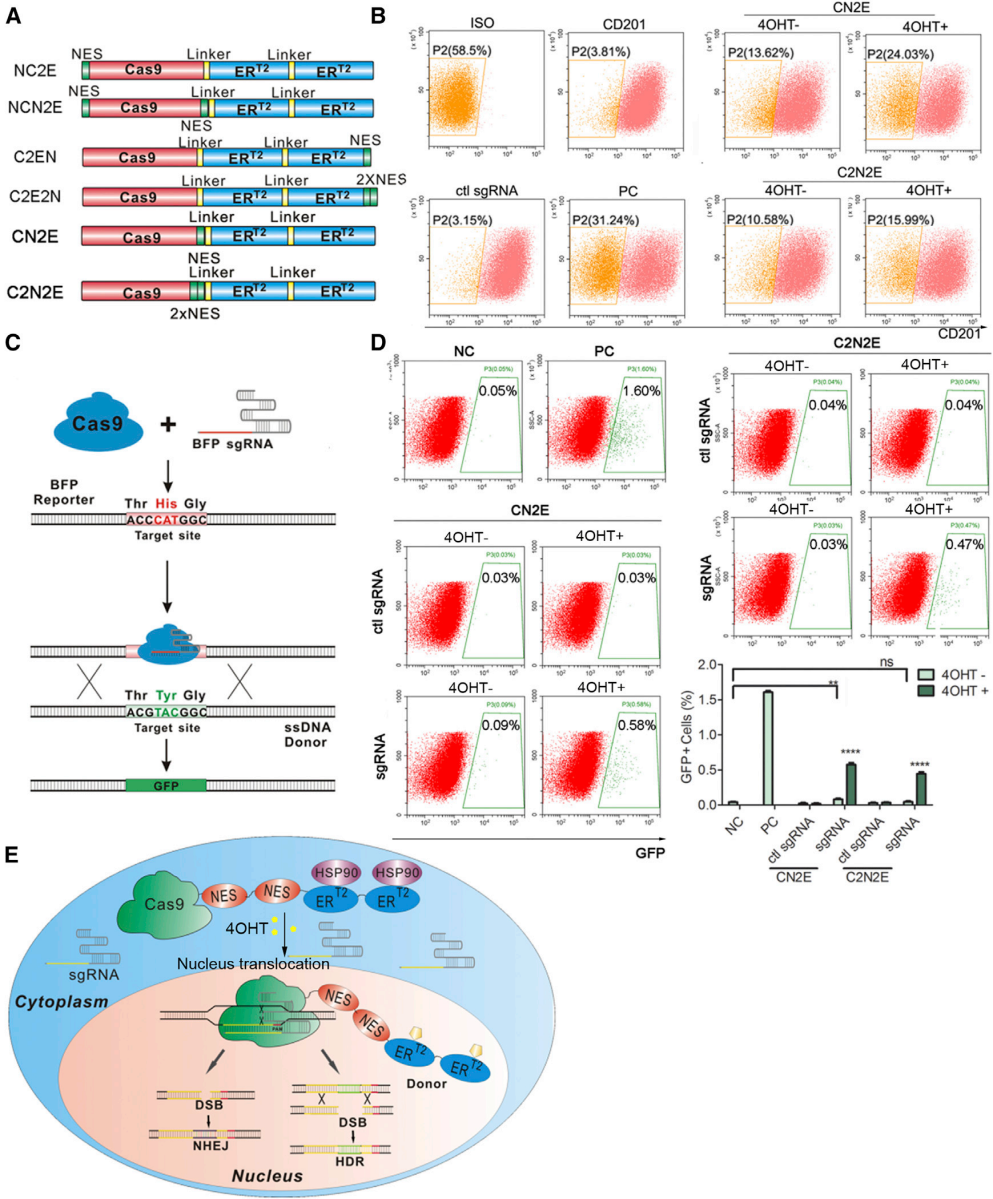
Introduction of NES Peptides to Lower Background Activity of HIT-Cas9 (A) Schematics (not drawn to scale) of various fusion constructs of NES-tagged Cas9-ER^T2^. (B) NHEJ-induced CD201 knockout was examined using flow cytometry. ISO, 293T cells incubated with antibody isotype control; CD201, 293T cells incubated with anti-CD201 antibody conjugated with PE-Vio770 fluorophore; ctl sgRNA, cells transfected with an unrelated sgRNA and incubated with the CD201 antibody; PC, cells transfected with Cas9-NLS and CD201 sgRNA and incubated with the CD201 antibody. (C) Cartoon illuminating the working mechanism of a fluorescence conversion reporter (FCR) assay, in which blue fluorescence protein (BFP) is converted to GFP upon HDR-mediated substitution of a key amino acid. (D) HDR efficiency was measured by flow cytometry in the FCR assay. Representative plots (top) and quantifications (right bottom) are shown. NC, cells transfected with an unrelated sgRNA; PC, cells transfected with Cas9-NLS and BFP sgRNA; ctl sgRNA, unrelated sgRNA. (E) Cartoon illuminating the working mechanism of the optimized drug-inducible HIT-Cas9 genome-editing system. Data showed mean ± SD. n = 3 biological replicates. ns, non-significant; **p < 0.01; ****p < 0.0001; Student’s t test.

The leakiness of the C2E construct is detrimental to its application. We hypothesized that the background activity might be caused by undesired nuclear localization in the absence of 4-OHT. Therefore, to damp its leakiness, we introduced one or two NES peptides to the C2E construct at various locations^14^, ^20^ (Figure 2A). Using the SSA luciferase assay, we found that Cas9 nuclease activity was best retained when a NES was inserted between Cas9 and 2ER^T2^ (Cas9-NES-2ER^T2^/CN2E) (Figure S4). Consistently, CN2E demonstrated efficient drug induction with no significant background activity in the TLR assays (Figure S5). On the contrary, we observed that NES at either terminus of C2E abolished its drug induction, indicating a critical requirement of the NES fusion site for optimal performance (Figures S4 and S5). NES also dramatically reduced background activity and delivered robust 4-OHT-dependent CD201 knockout (Figure S6). However, we noticed background activity in the CD201 assay not detected in previous TLR assays potentially due to the assay’s higher sensitivity. Accordingly, we sought to further reduce its background by insertion of two tandem NES peptides in between Cas9 and ER^T2^ domains (C2N2E). This indeed further lowered background activity while retaining drug inducible action, though compromised, in the CD201 knockout assay (Figure 2B). Considering low sensitivity of TLR assay to detect background activity (Figure S7), we adopted a fluorescence conversion reporter (FCR) assay,^21^ in which BFP is converted to GFP upon HDR mediated substitution of a key amino acid (Figure 2C). This assay demonstrated higher sensitivity than the TLR assays. It successfully detected background activity of the Cas9-NES-2ER^T2^ and its decrease upon insertion of an additional NES, although drug-induced activity was also compromised (Figure 2D). Taken together, our development of the HIT-Cas9 genome-editing system concluded at the C2N2E construct (Figure 2E).

**Figure 3:**
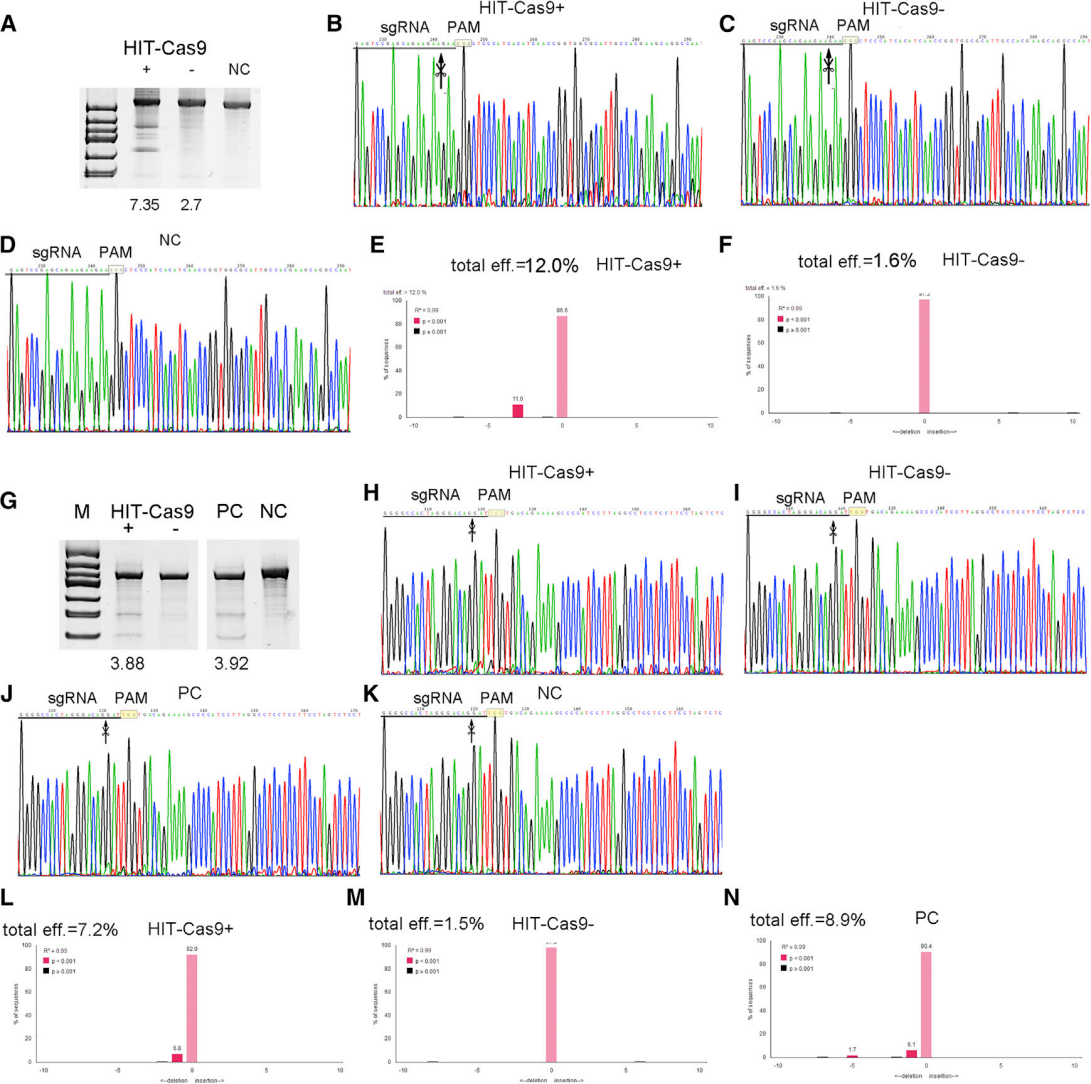
Drug-Inducible Genome Editing by HIT-Cas9 in Human ESCs and MSCs (A–F) Drug-inducible genome editing of an EMX1 site in a human ESC line H9 was examined in Surveyor assay (A) and TIDE assay (B–F). Sanger sequencing results upon 4-OHT treatment (B), without 4-OHT treatment (C), and of negative control (D) were shown. TIDE analyses were performed according to the instruction of a web tool (available at http://tide.nki.nl) upon 4-OHT treatment (E) and without 4-OHT treatment (F). (G–N) Drug-inducible genome editing of an AAVS1 site in primary MSCs derived from human adipose tissue was examined in Surveyor assay (G) and TIDE assay (H–N). Sanger sequencing results upon 4-OHT treatment (H), without 4-OHT treatment (I), of positive control (J), and of negative control (K) were shown. TIDE analyses were performed upon 4-OHT treatment (L), without 4-OHT treatment (M), and for the positive control (N). Percentages (%) of indel, if detected, were listed at the bottom of each lane for Surveyor analyses (A and G). Sanger sequencing results (B–D and H–K) and results from quantitative TIDE analyses (E and F, L–N) were shown. Expected cutting sites were labeled with arrows in Sanger sequencing results (B–D and H–K). M, marker; +/−, with or without 4-OHT; NC, cells transfected with HIT-Cas9 and an unrelevant sgRNA; PC, cells transfected with a NLS-tagged Cas9 and AAVS1 sgRNA.

Considering that such drug-inducible systems would find broad utility in targeting various cell types, among which human stem cells with clinical utilities would be of particular interest,^22^, ^23^, ^24^ we expanded our examination to additional human cell lines, including ESC line H9 (Figures 3A–3F), adipose-derived MSCs (Figures 3G–3K), and liver cancer cell line HepG2 (Figure S8). Notably, HIT-Cas9 and sgRNA constructs were delivered to H9 via lentiviral transfer. Both Surveryor assay and TIDE (tracking of indels by decomposition) assay^25^ confirmed its tight and effective drug inducibility across these cell types, thus suggesting a broad applicability of the HIT-Cas9 design. In addition, we also confirmed 4-OHT-induced nuclear translocation of this terminal optimized construct via immunofluorescence analysis against 3×Flag tags (Figure S9).

So far, we have used several assays, including the FCR assay, the TLR assay, the CD201 knockout assay, and the plasmid SSA (pSSA) assay, in optimization toward a tight and effective drug-inducible Cas9 construct. The higher sensitivity of the FCR assay than the TLR assay likely reflects higher HDR efficiency in point mutagenesis (FCR) than in longer DNA fragment exchange (TLR). In addition, mCherry signal in the TLR reporter that probes NHEJ events displayed a high background noise, consistent with its previous use after prior purification to remove mCherry-positive cells.^26^ Moreover, CD201 knockout assay, although quite sensitive in detection of NHEJ events, required prolonged culture for residue CD201 expression to decay and antibiotic enrichment for successfully transfected cells, which might bias the assay toward a high background noise. Finally, in the pSSA assay, without integration of the reporter construct to the genome, is not suitable to examine drug-inducible effects based on nuclear localization. Therefore, taking consideration of the pros and cons of all these assays, we decided to use the FCR assay as the primary system for further investigations, which is not only sensitive but also amenable to scaled applications.

### Comparison of HIT-Cas9 with Existing Designs

In pursuit of CRISPR/Cas9 technologies in greater precision, the field has reported several designs of drug-inducible systems previously.^10^, ^11^, ^12^, ^13^, ^14^, ^15^ An evolved intein, which undergoes 4-OHT-dependent splicing, was inserted within Cas9 at Serine 219 (intein-S219) and disrupts Cas9 function. Splicing triggered by 4-OHT restores Cas9 activity.^11^ In other designs, Cas9 was split into two halves, so called split-Cas9.^13^, ^14^ In one split system,^14^ each half was fused with a binding partner of mammalian target of rapamycin (mTOR), FK506 binding protein 12 (FKBP), and FKBP rapamycin binding (FRB) domain, respectively. Rapamycin induces binding of FKBP and FRB, thus bringing the two halves of Cas9 together and reconstituting its activity. Doxycycline-inducible Tet-on Cas9 systems were also reported, in which Cas9 activity was controlled at the transcription level and appeared to be slower in response to drug induction than those controlled at the post-translation level.^10^, ^12^, ^15^ Recently, an independent study identified that fusion of four ER^T2^ domains to Cas9 (iCas), two to each terminus, resulted in 4-OHT-inducible genome-editing activity. It was reported that this construct introduced a comparable background activity with intein and split systems but higher efficiency upon drug induction and a more rapid response to drug induction than Tet-on, intein, and split systems.^15^ We next benchmarked the drug-inducible performance of our system against these designs head-to-head in the same experiments in a carefully controlled fashion.

**Figure 4:**
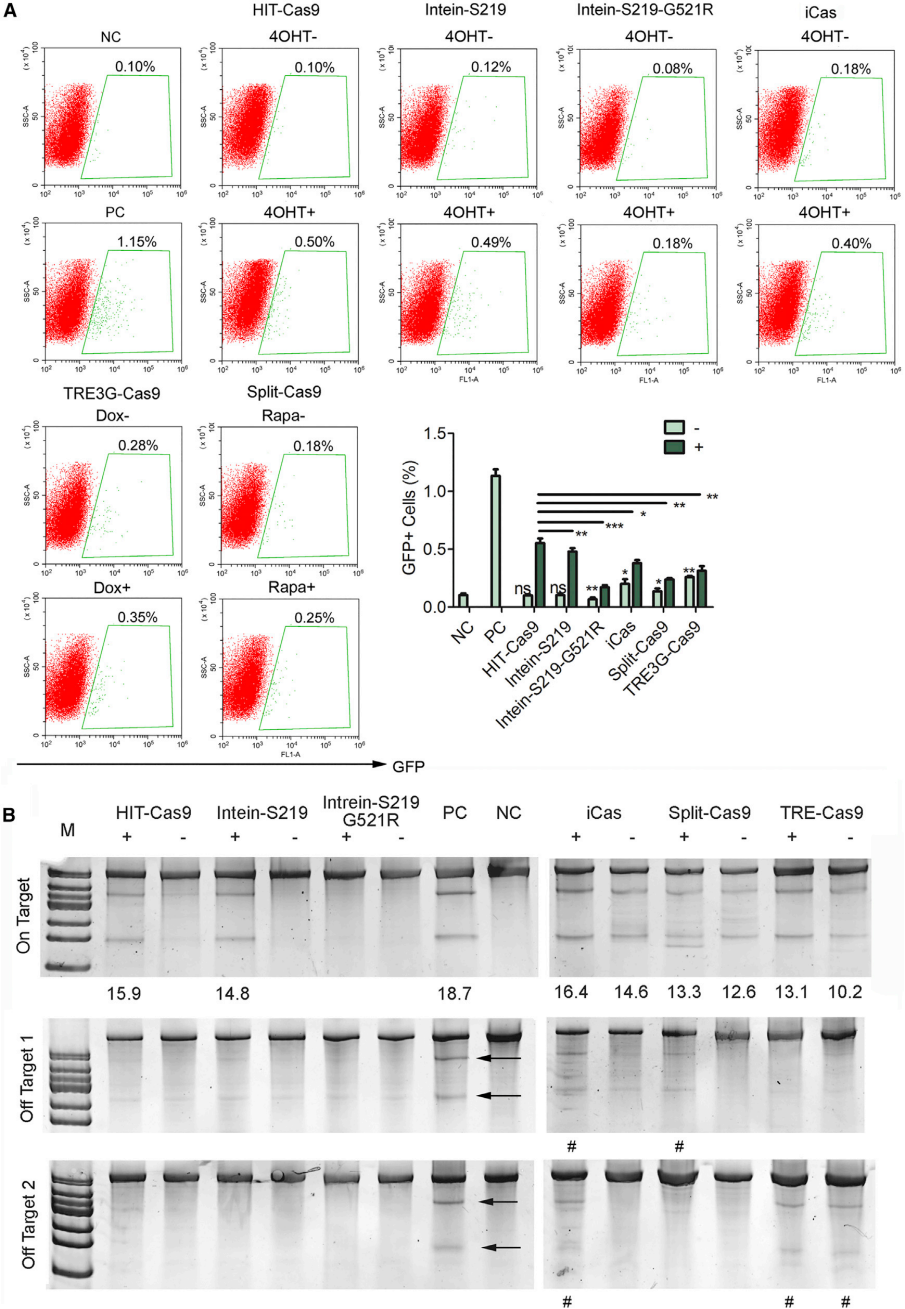
Comparison of HIT-Cas9 with Existing Drug-Inducible Designs (A) Comparative analyses of drug-inducible efficiency and background activity of HIT-Cas9 and previously reported designs using the FCR assay. Representative plots (top) and quantifications (right bottom) were shown. NC, cells transfected with an unrelated sgRNA; PC, cells transfected with Cas9-NLS and BFP sgRNA. Data showed mean ± SD. n = 3 biological replicates. ns, non-significant; *p < 0.05; **p < 0.01; ***p < 0.001; Student’s t test. (B) Drug-inducible genome editing of an EMX1 site and two off-target sites examined in Surveyor assay. Percentages (%) of indel, if detected, were listed at the bottom of each lane for on-target site analyses. Arrows point to off-target cleavage bands from NLS-Cas9. # denotes off-target activities for existing designs.

First, in the FCR assays, the HIT-Cas9 system delivered the highest editing activity in response to drug induction (Figure 4A). Meanwhile, significant background activities were observed from iCas, Split-Cas9, and tetracycline response element the 3rd generation (TRE3G)-Cas9, which utilized the latest generation of Tet-on system reported to deliver significantly reduced basal expression and increased sensitivity to doxycycline (https://clontech.com/US/Products/Inducible_Systems/Tetracycline-Inducible_Expression/Tet-On_3G) (Figure 4A). Among them, the leakiness of TRE3G-Cas9 was most pronounced, consistent with its use without doxycycline in previous independent studies.^27^, ^28^ On the contrary, no background activity was detected from HIT-Cas9 and Cas9 inserted with an intein (Intein-S219) or an intein mutant (G521R) (Figure 4A), in which the mutation renders it refractory to endogenous β-estradiol ligand, a property crucial for low background and selective control by the exogenous 4-OHT.^11^ Nonetheless, drug-induced activities of both intein systems were both significantly lower than the HIT-Cas9 (Figure 4A). Interestingly, Cas9 inserted with intein G521R mutant introduced a background activity lower than the basal level and its drug-induced activity was also dramatically lower than the other systems (Figure 4A).

To be less biased, we next further interrogated HIT-Cas9 in comparison with other systems by probing NHEJ events in Surveyor assays. All systems except the one utilizing the intein mutant (G521R) displayed similar on-target indel frequencies at an EMX1 site upon drug treatment (Figure 4B),^11^, ^15^ consistent with low HDR activity of the intein mutant (G521R) design in the FCR assay (Figure 4A). Notably, obvious background in the absence of drug induction were observed in iCas, Split-Cas9 and Tet-on designs (Figure 4A), a finding in agreement with results from the FCR assays (Figure 4A). Further, we also examined two known off-target sites for this sgRNA.^11^, ^15^ Positive control using an NLS-tagged Cas9 showed expected off-target activities (arrows in Figure 4B). Consistent with high background on-target cleavage from the iCas, Split-Cas9, and Tet-on systems, their off-target activities were detected at the off-target sites (# in Figure 4B), on contrary to the HIT-Cas9 and intein systems (Figure 4B). Taken together, in comparison with these drug-inducible constructs reported so far, HIT-Cas9 best retains on-target activity of Cas9 under tight drug control without introducing significant off-target effects. Notably, among the existing designs, Cas9 inserted with a wild-type intein also delivered tight and efficient drug-inducible HDR and NHEJ activities (Figure 4).

### Further Characterization of Sensitivity, Selectivity, and Speed of Response to 4-OHT Induction

In addition to the tightness and efficiency, an advantageous drug-inducible system should also be sensitive, selective, and rapid in response. We next further characterized HIT-Cas9 based on these additional criteria. In doing so, we used intein systems as comparison given that the rationale to utilize intein mutant is to increase selectivity of 4-OHT over β-estradiol.^11^ We also used iCas as comparison as it is also based on ER^T2^ regulation and it was reported to elicit a faster response than Tet-on, intein, and split systems.^15^

**Figure 5:**
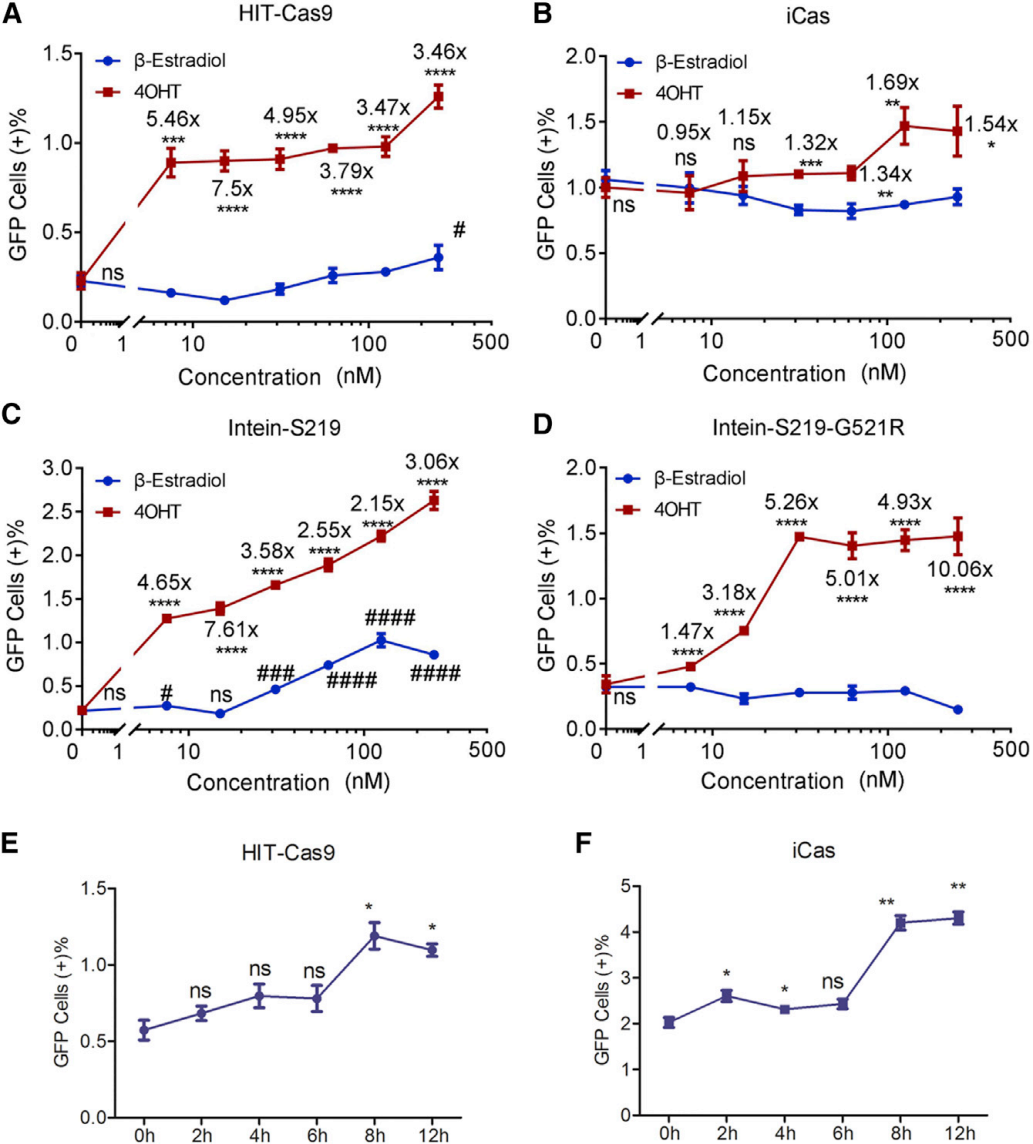
Comparative Analyses of Sensitivity, Selectivity, and Speed of Response to 4-OHT (A–D) Dose-dependent genome-editing activities of HIT-Cas9 (A), iCas (B), and two intein designs, Intein-S219 (C) and Intein-S219-G521R (D) examined using the FCR assay upon treatment with different concentration of β-estradiol or 4-OHT. (E and F) Time-lapse analyses of HIT-Cas9 (E) and iCas (F) in response to 400 nM 4-OHT using the FCR assay. Data showed mean ± SD. n = 3 biological replicates. ns, non-significant; *p < 0.05; **p < 0.01; ***p < 0.001; ****p < 0.0001; #p < 0.05; ###p < 0.001; ####p < 0.0001; Student’s t test. Fold of activation by 4-OHT over the same concentration of β-estradiol was displayed (A–D).

We first examined dose dependent response of the optimized terminal HIT-Cas9 system to 4-OHT and β-estradiol respectively. Sensitive and selective response of HIT-Cas9 to 4-OHT over β-estradiol was observed in the FCR assay (Figures 5A and S10). An efficient induction of 4-OHT starts at nano-molar concentration. In strong contrast, a statistically significant activity was only detected when 250 nM β-estradiol was administrated, an indication of high selectivity. iCas showed selective response to 4-OHT, while its folds of induction in comparison with β-estradiol at the same concentrations were markedly lower, possibly due to its high background level (Figures 5B and S10). As expected, only intein harboring the G521R mutation, not the wild-type, delivered selective response to 4-OHT (Figures 5C, 5D, and S10). However, Cas9 inserted with the intein mutant required a much higher concentration of 4-OHT than HIT-Cas9 to effectively turn on its activity (Figures 5A, 5D, and S10). Taking these results together (Figures 5A–5D and S10), HIT-Cas9 remains advantageous in terms of sensitivity and selectivity to 4-OHT over the endogenous ligand, β-estradiol.

iCas was reported to act at a higher speed than intein, split, and Tet-on constructs in response to drug induction.^15^ We next sought to determine the paralleled course of editing events for iCas and HIT-Cas9 constructs upon drug induction. An obvious increase of fluorescent conversion in FCR assay starting at 8 hr of 4-OHT induction was observed for both HIT-Cas9 and iCas, an indication of inducible response at a similar speed (Figures 5E, 5F, and S11). Collectively, taking in account comparative analyses in this study and others,^10^, ^11^, ^12^, ^14^, ^15^ advantageous performances of our HIT-Cas9 system can be established according to multiple criteria of drug-inducible Cas9 modulation, including low background, high efficiency, low off-target effects, high sensitivity, high selectivity, and rapid response.

### Adoption of the HIT-Cas9 Architecture to Orthogonal *Staphylococcus aureus* Cas9

**Figure 6:**
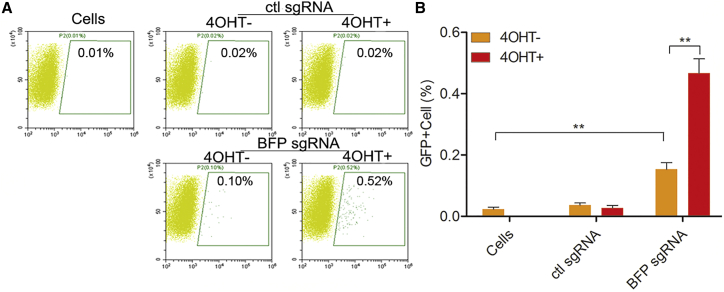
Adoption of the HIT-Cas9 Architecture to SaCas9 Drug-inducible genome editing by SaCas9-2NES-2ER^T2^ was examined in the FCR assay. Representative plots (A) and quantifications (B) were shown. Data showed mean ± SD. n = 3 biological replicates. ns, non-significant; **p < 0.01; Student’s t test.

The current study is based on the prevalent *Streptococcus pyogenes* (SpCas9). Orthogonal Cas9 species have distinct PAM requirements, thus expanding target coverage in the genome.^5^ *Staphylococcus aureus* Cas9 (SaCas9) has been of considerable interest as an orthogonal Cas9 species due to high activity in mammalian cells, the high incidence of its PAM sequence (NNGRR), and its smaller protein size, a crucial property for the development of gene therapy because of restrictive cargo sizes for certain delivery vehicles such as adeno-associated virus (AAV).^29^ Therefore, we next sought to adopt our optimized HIT-Cas9 architecture to the SaCas9 species. To this end, we generated a SaCas9-2NES-2ER^T2^ construct for drug-inducible genome editing. When delivered with a sgRNA targeting the same region on BFP and compatible with SaCas9, this construct managed to convert the fluorescence to GFP. It showed robust drug-inducible regulation dependent on a specific sgRNA, albeit higher background activity than that of SpCas9, a feature that warrants further optimization (Figure 6). These results suggest a potentially generalizable architecture of HIT-Cas9, thus further expanding its applicability.

## Discussion

In summary, we have engineered HIT-Cas9, an advantageous drug-inducible CRISPR/Cas9 device for genome editing upon vigorous optimization and characterization. In the HIT-Cas9 modular device, fusion of two NES peptides and two ER^T2^ domains to Cas9 at its C terminus enables efficient drug-inducible genome editing via both NHEJ and HDR with minimal background (Figure 2, 3, S7, and S8). Cross-comparisons with multiple existing designs, including intein, split, Tet-on, and iCas,^10^, ^11^, ^12^, ^13^, ^14^, ^15^ demonstrated better performances of the HIT-Cas9 system according to several criteria for a good drug-inducible system (Figures 4, 5, S10, and S11). Notably, iCas was also developed based on engrafting ER^T2^ to Cas9. Its high background activity might be derived from a large bulk of protein fusions with four ERT2 domains and a NLS tag, which might trap the protein into the nucleus. Its lower efficiency in response to 4-OHT treatment might be because of less retained Cas9 activity by N-terminal fusion that was observed in the beginning of this study (Figures S1C, S1E–S1G, and S2). On the contrary, HIT-Cas9 is more compact, and the use of NES was demonstrated to be critical in controlling its background activity (Figures 2, S6, and S7). Furthermore, even in a race for the higher speed of drug induction, which was reported as a major advantage of iCas over other systems,^15^ our results suggest a tie (Figures 5E, 5F, and S11). In addition to the existing drug-inducible systems examined in this study, a latest report deployed ER^T2^ domain on top of the split design to reduce its background activity, consistent with our observations supporting ER^T2^ as a tight regulator of drug induction.^30^ Last but not least, successful adoption of HIT-Cas9 architecture to SaCas9 demonstrated potential further expansion of its applicability (Figure 6).

It is worthy pointing out that, in comparison with the standard Cas9 tagged with NLS included as positive controls (PC), compromised activity was observed upon drug induction for multiple drug-inducible CRISPR/Cas9 systems in our hands and others (Figure 4).^11^, ^13^, ^14^, ^15^, ^30^ This suggests that subjecting the CRISPR/Cas9 system to drug control, independent of the working mechanisms, commonly compromises its activity in comparison to a constitutive system that deliver effector agents to the maximum level. Future investigations might be able to further enhance the drug-induced activity. And compromising activity in exchange with control in greater precision (e.g., less off-target activities shown in Figure 4B) would be worthwhile in certain applications such as gene therapy, where safety is a top-priority concern.

Above all, continuous development toward greater precision in using drugs to control biological events is always desired for biomedical research and potential clinical applications in a safer and more effective manner. Temporal control and dose-dependent control of the CRISPR/Cas9 machinery using HIT-Cas9, together with other HIT devices we have reported recently for transcriptional programming based on CRISPR/Cas9 and those based on transcription activator-like (TAL) effectors,^31^, ^32^ would open broad avenues toward many applications as a comprehensive toolbox. Further optimization of the these designs and future development of additional systems using engineered SpCas9 with distinct PAM requirements^33^ or Cas9 from other species^5^ will further enhance their performances and expand the repertoire of genomic loci they can modify.

## Materials and Methods

### Plasmid Construction

Cas9 and ER^T2^ were cloned from the pX330-U6-Chimeric_BB-CBh-hSpCas9 plasmid (a gift from Feng Zhang, Addgene plasmid #42230^2^) and the pAd-CreER plasmid (a gift from T.C. He’s lab, Chicago University), respectively. NES sequences^34^ were synthesized (Sangon Biotech) and cloned into Cas9 constructs. TRE3G Tet-on constructs were cloned based on the Tet-On 3G-inducible expression system from Clontech. Intein-S219 and intein-S219-G521R were obtained from Addgene and gifts from David Liu (Addgene plasmid #64190^11^; Addgene plasmid #64192^11^). Split-Cas9 was obtained from Addgene and a gift from Feng Zhang (Addgene plasmid #62889^14^). SaCas9-2NES-2ER^T2^ was cloned by replacing SpCas9 with SaCas9 (a gift from Feng Zhang; Addgene plasmid #61591^29^). The HIT-Cas9 construct reported in this study will be available through Addgene.

To identify effective sgRNA candidates, we employed the genetic perturbation platform (GPP) web portal (https://www.broadinstitute.org/rnai/public/analysis-tools/sgrna-design)^35^, ^36^ and the CRISPR DESIGN web portal (http://crispr.mit.edu/)^37^ to predict high-efficiency and low off-target effect by the computational analyses. sgRNAs are cloned into an optimized sgRNA scaffold (A-U flip extension)^27^ (Table S1). The BFP sgRNA used with SaCas9 was identified based on PAM sequence requirement (NNGRRT)^29^ and also cloned into an optimized sgRNA scaffold (A-U flip)^38^ (Table S3).

To generate pSSA reporter plasmids, sgRNA target sequences were cloned in between the homology domains of a gaussia luciferase reporter.^17^ To generate the TLR plasmid used in this study, Sce target site was replaced by the target sequence of human Oct4 sgRNA-3 (Figure S1D) in the plasmid of pCVL Traffic Light Reporter 1.1 (Sce target) Ef1a Puro (a gift from Andrew Scharenberg, Addgene plasmid #31482).^19^ GFP donor plasmid used in conjunction was obtained from Addgene and a gift from Andrew Scharenberg (Addgene plasmid #31475).^19^

### Cell Culture

HEK293T and HepG2 cells (American Type Culture Collection [ATCC]) were cultured in DMEM supplemented with 10% fetal bovine serum (FBS), 2 mM GlutaMAX (Thermo Fisher), 100 U/mL penicillin, and 100 μg/mL streptomycin at 37°C and with 5% CO_2_. Human adipose-derived MSCs were purchased from the Shanghai Zhong Qiao Xin Zhou Biotechnology and maintained in DMEM: Nutrient Mixture F-12 (DMEM/F-12) medium supplemented with 10% FBS at 37°C and with 5% CO_2_. ESC line H9 was purchased from Wicell Research Institute and maintained in a 6-well plate coated with Matrigel (BD Biosciences) in Essential 8 medium (Thermo Fisher).

HIT-Cas9 and EMX1 sgRNA were cloned in lentiviral expression plasmids, packaged in lentiviral particles, and delivered to H9 cells. The cells were then treated with 2.5 μg/mL of Zeocin (InvivoGen), whose drug resistance cassette was co-expressed with HIT-Cas9, and 0.5 μg/mL Puromycin (InvivoGen), whose drug resistance cassette was co-expressed with sgRNA, to enrich cells that were successfully infected by both constructs. An unrelevant sgRNA was used as a negative control. 3 days post-antibiotic selection, 4-OHT was added into the medium to the working concentration at 200 nM. Genomic DNA was isolated and applied for surveyor assay and TIDE analyses 4 days upon 4-OHT treatment.

As for MSCs, HIT-Cas9 and AAVS1 sgRNA constructs were packed in polyethyleneimine (PEI)-based lipid particles and transfected following standard protocol. A plasmid that constitutively expresses GFP was co-transfected into the MSCs. Two days after 4-OHT induction, GFP-positive cells were harvested by fluorescence-activated cell sorting (FACS) and genomic DNA was isolated for surveyor assay and TIDE analyses.

TLR and FCR reporter constructs was integrated into the genome via lentiviral infection. Monoclonal cell lines were obtained respectively. Transfections were conducted with Biotool DNA transfection reagent (Biotool) following standard protocol. To ensure proper controlled comparison, the molar amount of Cas9, sgRNA, and donor as well as the total weight of transfected DNA were matched for each well in all experiments. Unless stated otherwise, 125 nM of 4-OHT or a matched volume of ethanol was added to culture medium 5 hr after transfection, and treatments lasted for 48 hr.

### pSSA assay

pSSA reporter, sgRNA, and Cas9 constructs and an internal control construct that constitutively expresses firefly luciferase were co-transfected into HEK293T cells. Medium was collected 48 hr after transfection and gaussia luciferase substrate (New England Biolabs) was added. Cells were lysed and firefly luciferase substrate (Promega) was added. Luciferase readouts were measured using VICTOR *X*3 Multilabel Plate Reader (PerkinElmer) according to the manufacturer’s recommended protocol.

### TLR Assay

TLR reporter cell line were pre-seeded and transfected with various Cas9 constructs and sgRNA with or without GFP donor template (Addgene plasmid #31475^19^). Flow cytometry analysis of GFP-positive cells was performed using CytoFLEX cell analyzer (Beckman Coulter) to assess HDR efficiency. At least 50,000 cells from each well were analyzed. To assess NHEJ efficiency, mCherry signal was analyzed using Harmony 3.5 (PerkinElmer) after image acquisition with Operetta High Content Screening system (PerkinElmer).

### CD201 Genomic Knockout Assay

HEK293T cells were cultured and transfected using standard protocols. 24 hr after transfection, 125 μg/mL Zeocin and 100 μg/mL G418 were applied to enrich transfected cells. 4-OHT was added 48 hr post-transfection. Upon reaching sufficient number for flow cytometry analyses, cells were collected and stained live with a CD201 antibody conjugated with PE-Vio770 (MiltenyiBiotec). Flow cytometry analysis was conducted using the CytoFLEX (Beckman Coulter).

### FCR Assay

FCR stable cell line was co-transfected with various Cas9 constructs and BFP sgRNA and single strand DNA (ssDNA) donor template. The sequence of donor was 5′-GCCACCTACGGCAAGCTGACCCTGAAGTTCATCTGCACCACCGGCAAGCTGCCCGTGCCCTGGCCCACCCTCGTGACCACCCTGACGTACGGCGTGCAGTGCTTCAGCCGCTACCCCGACCACATGA-3′^21^ (synthesized by Sangon Biotech). To measure HDR efficiency, flow cytometry analysis for GFP-positive cells was conducted using CytoFLEX cell analyzer (Beckman Coulter). 30,000 cells at minimum from each well were analyzed.

### Subcellular Localization Analyses

GFP or 3×Flag tagged Cas9 constructs were transfected into HEK293T Standard immunofluorescence staining protocol was followed to label 3×Flag epitopes. Images were acquired and quantitatively analyzed using Operetta High Content Screening system (PerkinElmer) after cells being fixed by 4% (w/v) paraformaldehyde and stained with Hochest 33342 (Thermo Fisher).

### Surveyor and TIDE Assays

Genomic DNA from various types of cells delivered with different Cas9-ER^T2^ fusion constructs and sgRNAs targeting either the EMX1 gene or AAVS1 site (Table S1) was extracted using wizard genomic DNA purification kit (Promega). PCR was performed to amplify target and off-target loci from 100 ng of genomic DNA using a high-fidelity polymerase (Accuprime Taq Hifi from Thermo Fisher) in 25 μL reactions. The sequences of primers were listed in Table S2. A mixture containing 5 μL of PCR products and 4.5 μL of 1× Accuprime buffer II was then denatured by heating to 95°C and slowly reannealed from 95°C to 85°C by 2°C/s followed by 0.1°C/s from 85°C to 25°C for rehybridization using Mastercycler nexus gradient (Eppendorf). Samples were then incubated with Surveyor nuclease (Transgenomic) for 20 min at 42°C. The nuclease recognizes and cleaves DNA mismatches (wild-type:mutant hybridization). The digested products were electrophoresed through a 15% acrylamide gel and visualized by EB staining. Quantification of cleavage bands was performed using ImageJ software (NIH). The genome cleavage efficiency was calculated by the following formula:

$$\mathrm{Indel}\;\mathrm{percentage}\;\left(\%\right)=100\;x\;\left(1–{\left(1-\mathrm{fraction}\;\mathrm{cleaved}\right)}^{1/2}\right).$$

The PCR products were also sequenced for TIDE analyses in some experiments to assess genome editing by HIT-Cas9 using a web tool (available at https://tide.nki.nl/).

## Author Contributions

Y.W. conceived and supervised the study. C.Z., Y. Zhao, J.Z., J.L., L.C., Y. Zhang, Y.Y., and Y.W. designed experiments. C.Z., Y. Zhao, J.Z., J.L., L.C., Y. Zhang, Y.Y., and S.W. performed experiments. C.Z., Y. Zhao, J.Z., J.L., L.C., Y. Zhang, Y.Y., J.X., and Y.W. analyzed data. C.Z., Y. Zhao, J.Z., J.L., L.C., and Y.W. wrote the manuscript.

## Conflicts of Interest

Patents covering the novel designs in this work have been filed.

## Supplemental Information

Document S1. Figures S1–S11 and Tables S1–S3

Document S2. Article plus Supplemental Information
