# Supplementary material for: HIT-Cas9: A CRISPR/Cas9 Genome-Editing Device under Tight and Effective Drug Control
Source: Mol Ther Nucleic Acids. 2018 Sep 1;13:208–19. doi: 10.1016/j.omtn.2018.08.022 (PMC6178243; doi:10.1016/j.omtn.2018.08.022)
Supplement: Document S1. Figures S1–S11 and Tables S1–S3 [file mmc1.pdf]

OMTN, Volume *13*

Supplemental Information

HIT-Cas9: A CRISPR/Cas9 Genome-Editing Device under Tight and Effective Drug Control

Chen Zhao, Yingze Zhao, Jingfang Zhang, Jia Lu, Li Chen, Yue Zhang, Yue Ying, Junjun Xu, Shixian Wei, and Yu Wang

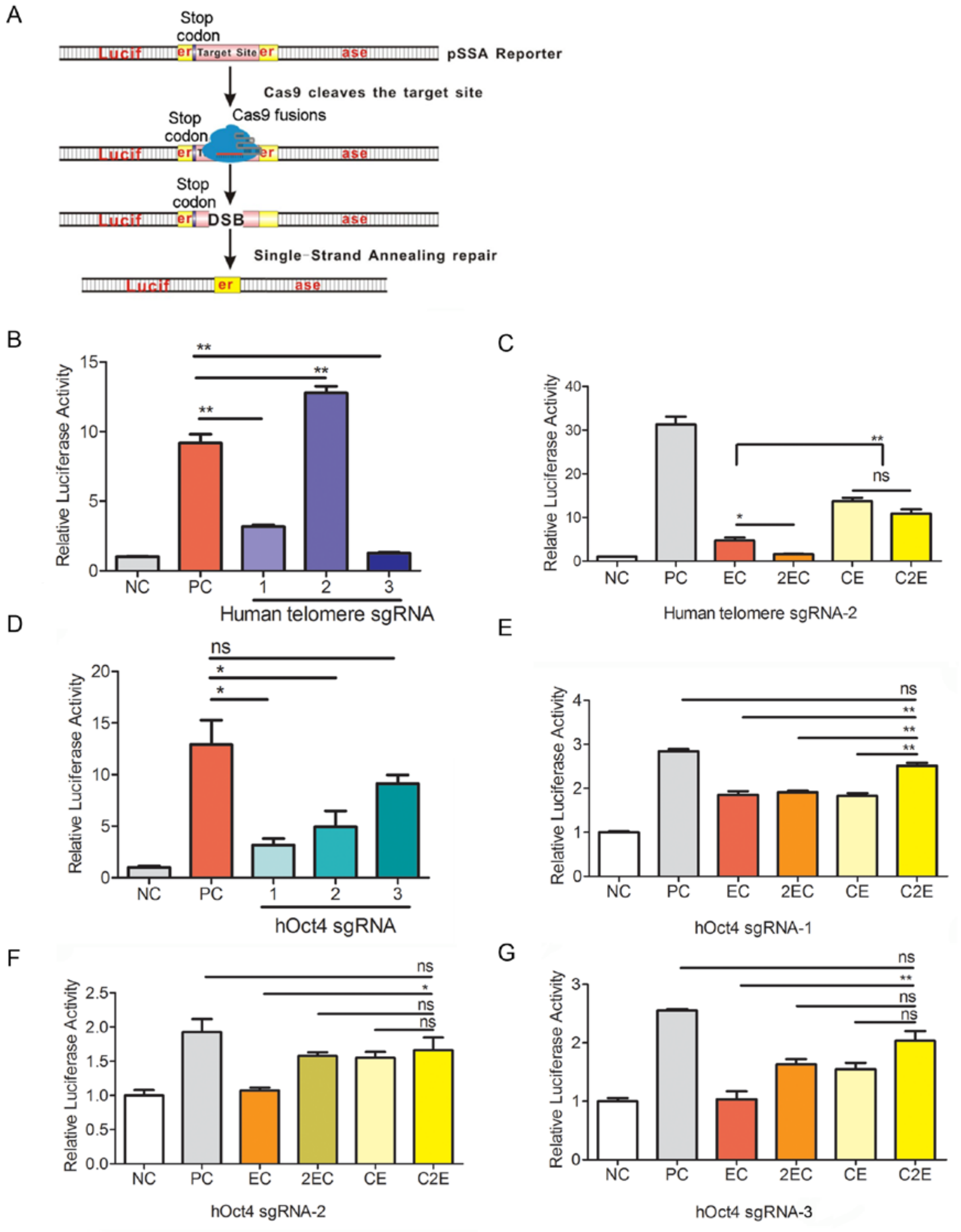

Supplementary Figure 1. Design and characterization of ER^{T2} fused Cas9 constructs. (A) Cartoon illustrating the working mechanism of the pSSA luciferase reporter assay. **(B)** The activity of three single chimeric guide RNAs (sgRNAs) targeting human telomere were examined using the Cas9-NLS construct in 293T cells. Cells transfected with a control sgRNA targeting the sequence flanking inserted site was used as positive control (PC). **(C)** The examination of DNA cleavage activity of different Cas9 constructs using

human telomere sgRNA-2. **(D)** The activity of three single chimeric guide RNAs (sgRNAs) targeting human Oct4 gene were examined using Cas9-NLS construct.**(E-G)** The DNA cleavage activity of different Cas9 constructs was examined using sgRNAs targeting human Oct4 gene. Cells transfected with the same amount of reporter construct while keeping the total amount of transfection constant were used as negative controls (NC). Cells transfected with a control sgRNA targeting the sequence flanking the target insertion site was used as positive control (PC). Data showed mean ± SD. n=3 biological replicates. ns: non-significant;*p<0.05; **p<0.01; student t test.

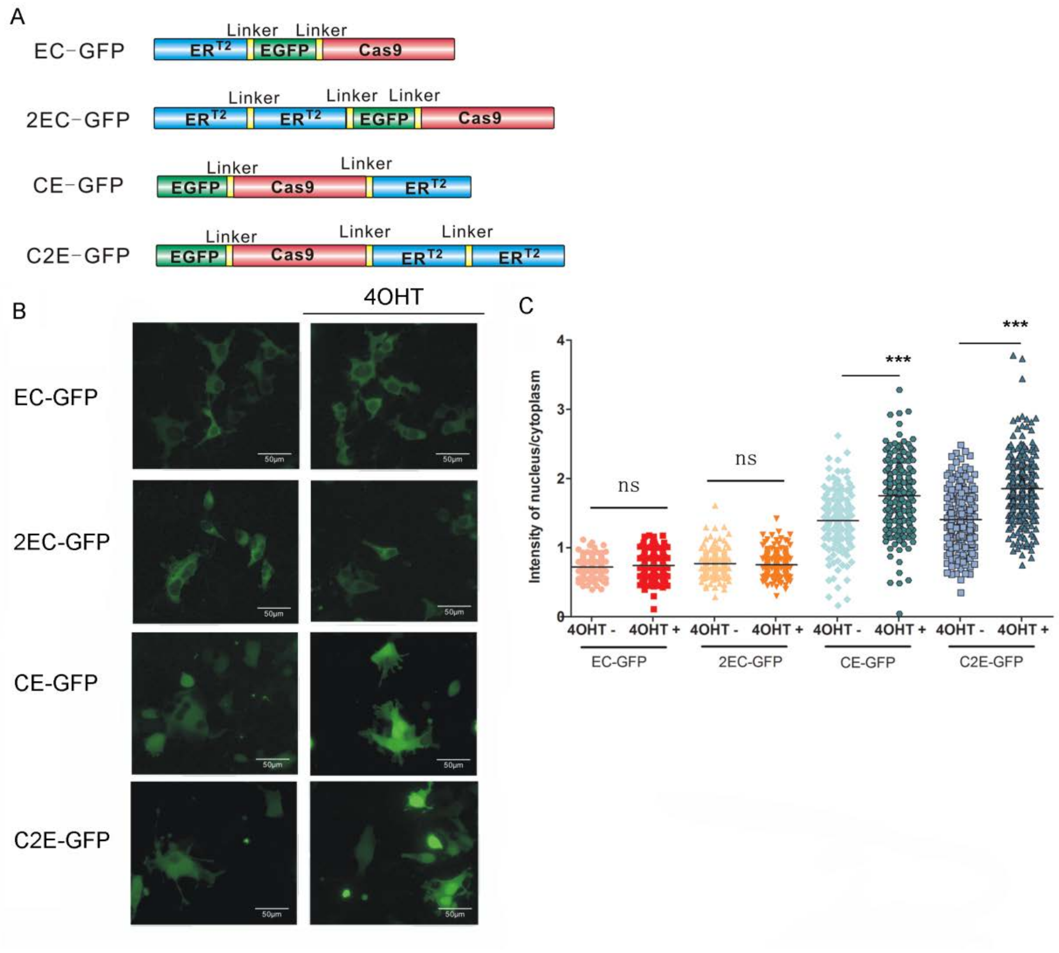

Supplementary Figure 2. Subcellular localization analyses of various ER^{T2} fusion constructs of GFP tagged Cas9 in response to 4-OHT treatments. (A) Schematics of various fusion constructs of GFP tagged Cas9-ER^{T2}. **(B)** Representative images of GFP from these fusion constructs with/without 4-OHT. **(C)** Quantitative analyses on the ratio of nucleus/cytoplasm GFP intensity.150-300 cells per condition were analyzed. Data showed mean ± SD. ns: non-significant; *** $p<0.001$; student t test.

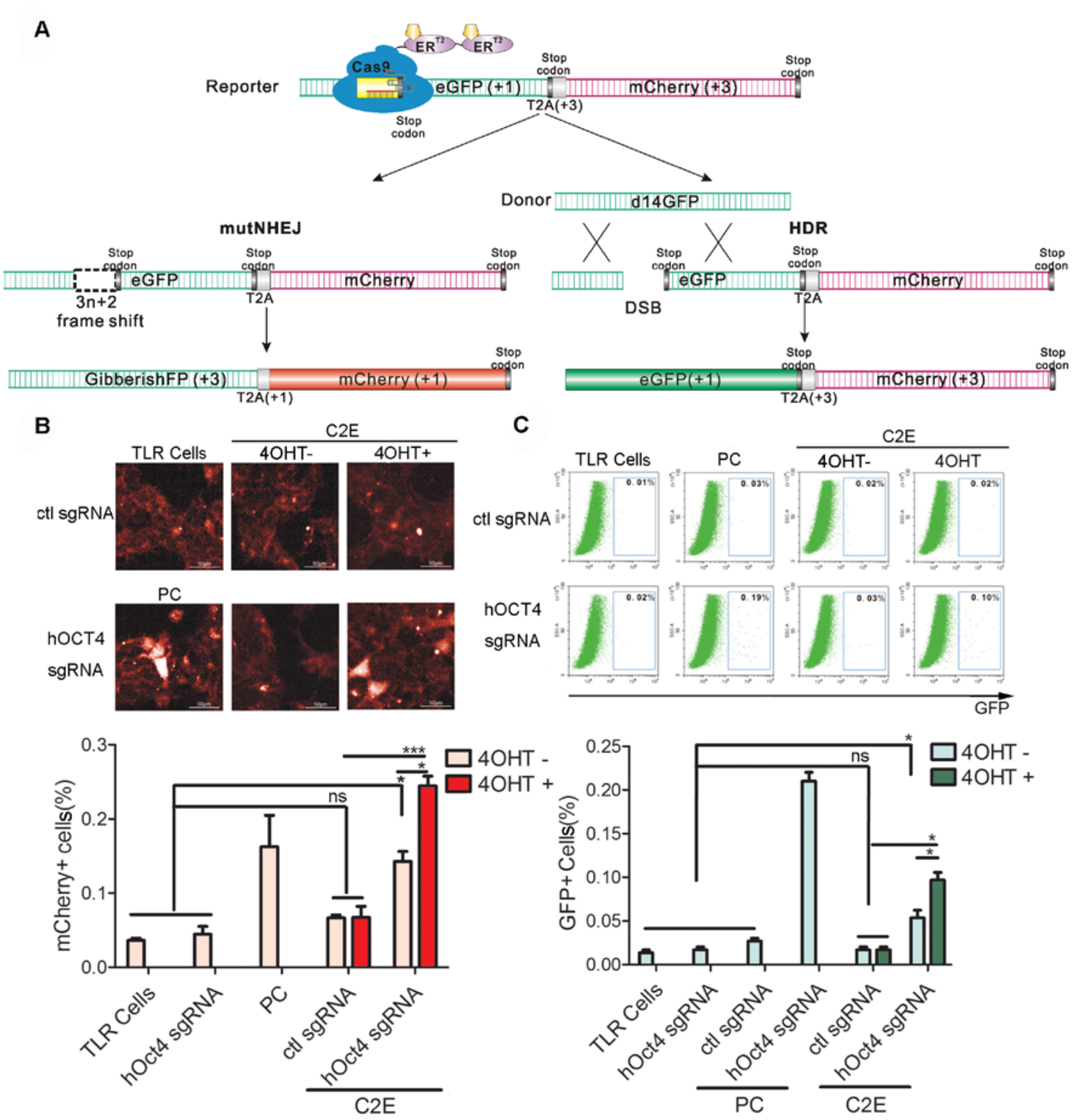

Supplementary Figure 3. Examination of C2E using the traffic light reporter (TLR). **(A)** Cartoon illuminating the mechanism of TLR. **(B)** NHEJ efficiency was examined with mCherry fluorescence using theTLR-Oct4 cell line. Representative images (upper panels) and quantifications (lower panel) were shown. **(C)** HDR efficiency was examined with GFP fluorescence using flow cytometry in the TLR-Oct4 cell line. Representative plots (left panels) and quantifications (right panel) were shown. Cells transfected with Cas9-NLS plasmid were used as positive control (PC). Data showed mean ± SD. n=3 biological replicates. ns: non-significant;*p<0.05; *** p<0.001; student t test.

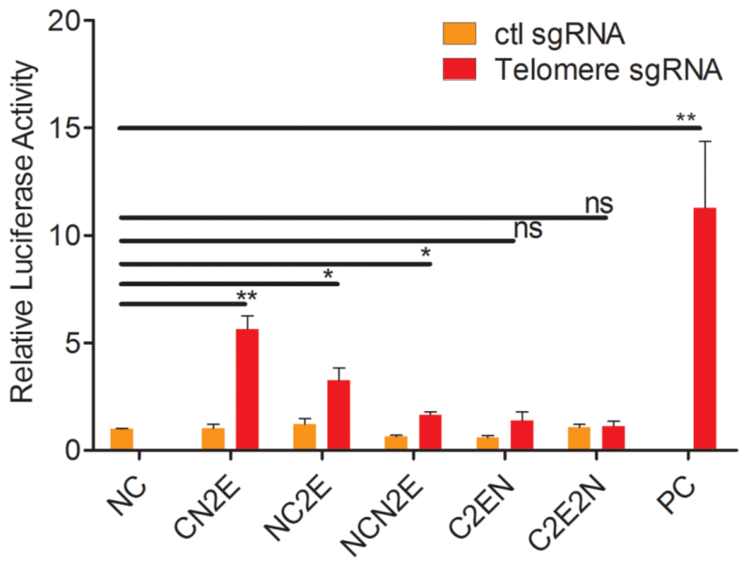

Supplementary Figure 4. The DNA cleavage activity of Cas9-2ER^{T2} (C2E) constructs tagged with NES peptides. The DNA cleavage activity of various Cas9 constructs was examined using the pSSA luciferase reporter containing the target site of telomere sgRNA-2 shown in Suppl. Fig. 1B. Cells transfected with Cas9-NLS was used as positive control (PC). Cells transfected with the same amount of reporter construct while keeping the total amount of transfection constant were used as a negative control (NC). Data showed mean ± SD. n=3 biological replicates. ns: non-significant;*$p<0.05$; **$p<0.01$; student t test.

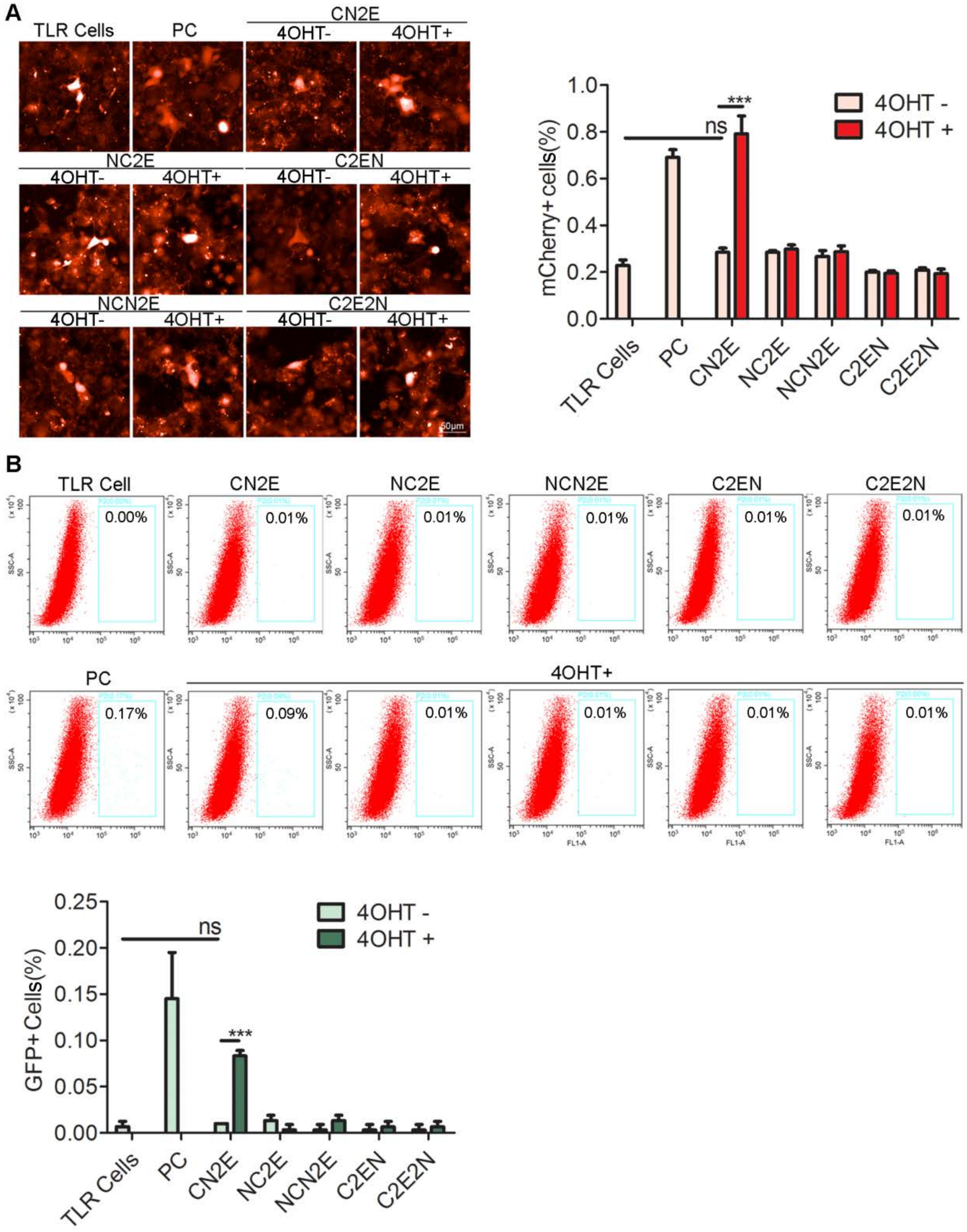

Supplementary Figure 5. Drug inducible gene editing using various NES tagged C2E constructs examined in the TLR assays.(A) NHEJ efficiency examined with mCherry fluorescence using the TLR-Oct4 cell line. Representative images (left panels) and quantifications (right panel) were shown. **(B)** HDR efficiency examined with GFP fluorescence using flow cytometry in the TLR-Oct4 cell line. Representative plots (upper panels) and quantifications (lower panel) were shown. Cells transfected with Cas9-NLS plasmid were used as positive control (PC). Data showed mean ± SD. n=3 biological

replicates. ns: non-significant;*** $p<0.001$; student t test.

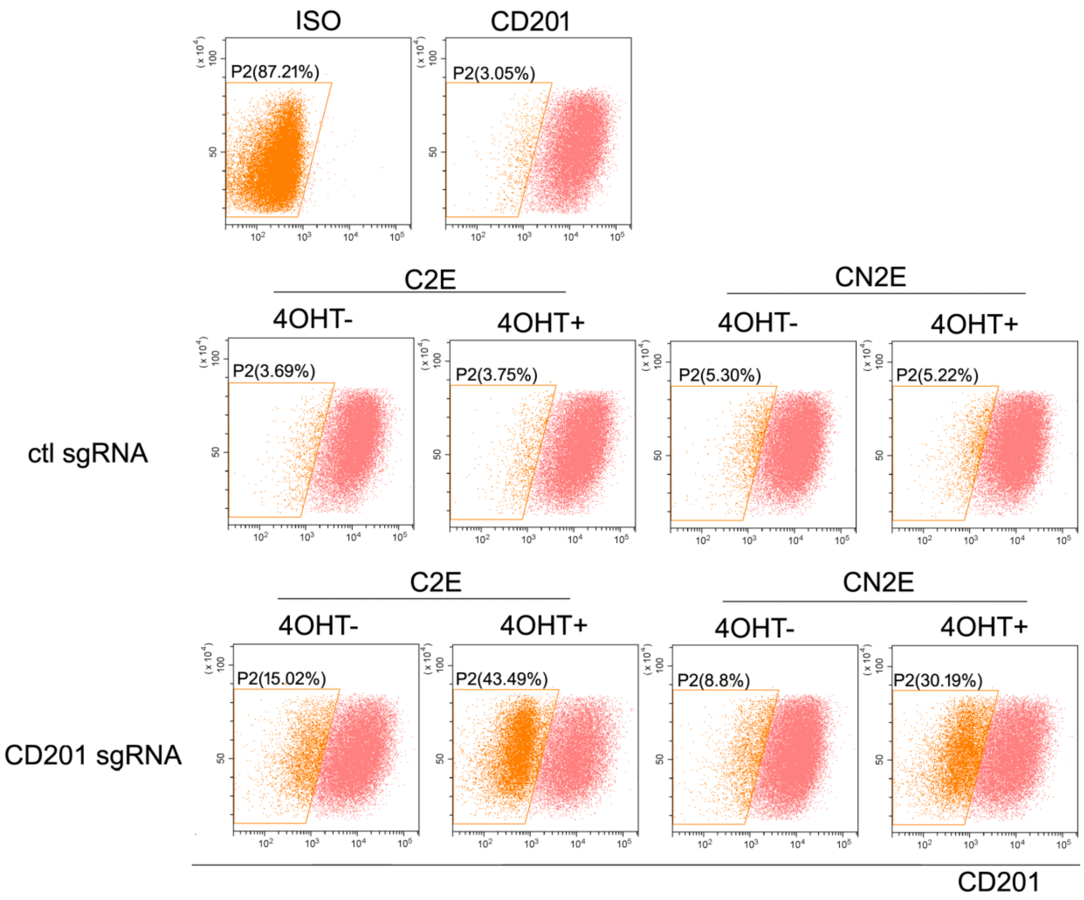

Supplementary Figure 6. CD201 knockout using C2E and CN2E. Representative plots showed the percentage of CD201 negative cells with/without the induction of 4-OHT. ISO, 293T cells incubated with antibody isotype; CD201, 293T cells incubated with anti-CD201 antibody.

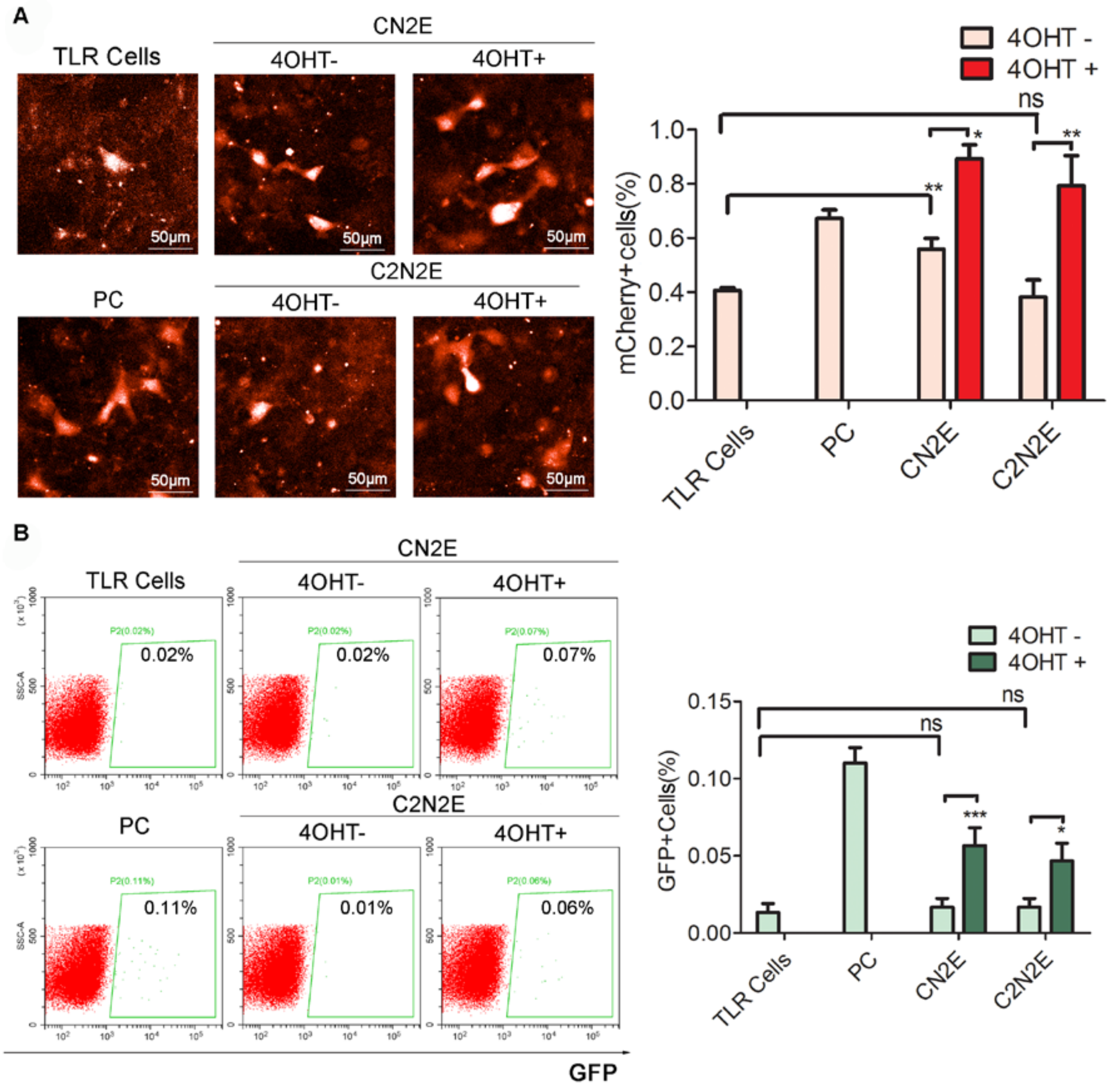

Supplementary Figure 7. C2E constructs inserted with one or two NES peptides examined in TLR assays. (A) NHEJ efficiency examined with mCherry fluorescence using the TLR-Oct4 cell line. **(B)** HDR efficiency examined with GFP fluorescence using flow cytometry in the TLR-Oct4 cell line. Representative images (left panels) and quantifications (right panel) were shown. Cells transfected with Cas9-NLS plasmid were used as positive control (PC).Data showed mean ± SD. n=3 biological replicates. ns: non-significant;*p<0.05; **p<0.01; *** p<0.001; student t test.

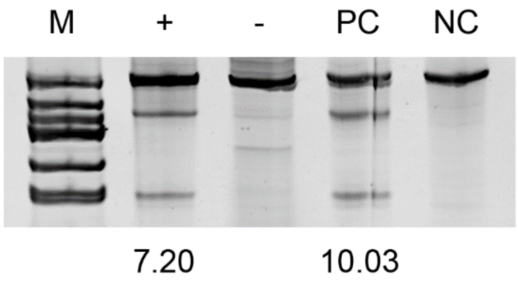

Supplementary Figure 8. Drug inducible genome editing of an EMX1 site in hepG2 cells using HIT-Cas9 was examined in Surveyor assay. Percentages (%) of indel, if detected, were listed at the bottom of each lane. M: marker; +/-, with or without 4-OHT; PC, cells transfected with Cas9-NLS and EMX1 sgRNA; NC, cells transfected with Cas9-NLS and an unrelated sgRNA.

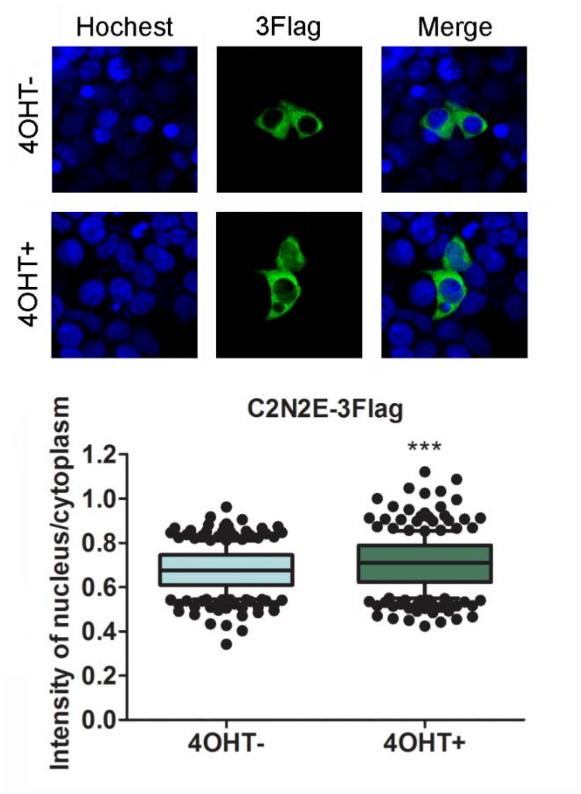

Supplementary Figure 9. Subcellular localization of 3 × Flag tagged Cas9-2NES-2ER^{T2} (C2N2E) with the induction of 4-OHT. Representative images (upper panels) and quantification analyses (bottom panel) were shown. Over 300 cells per condition were analyzed. Data showed mean ± SD. *** $p<0.001$; student t test.

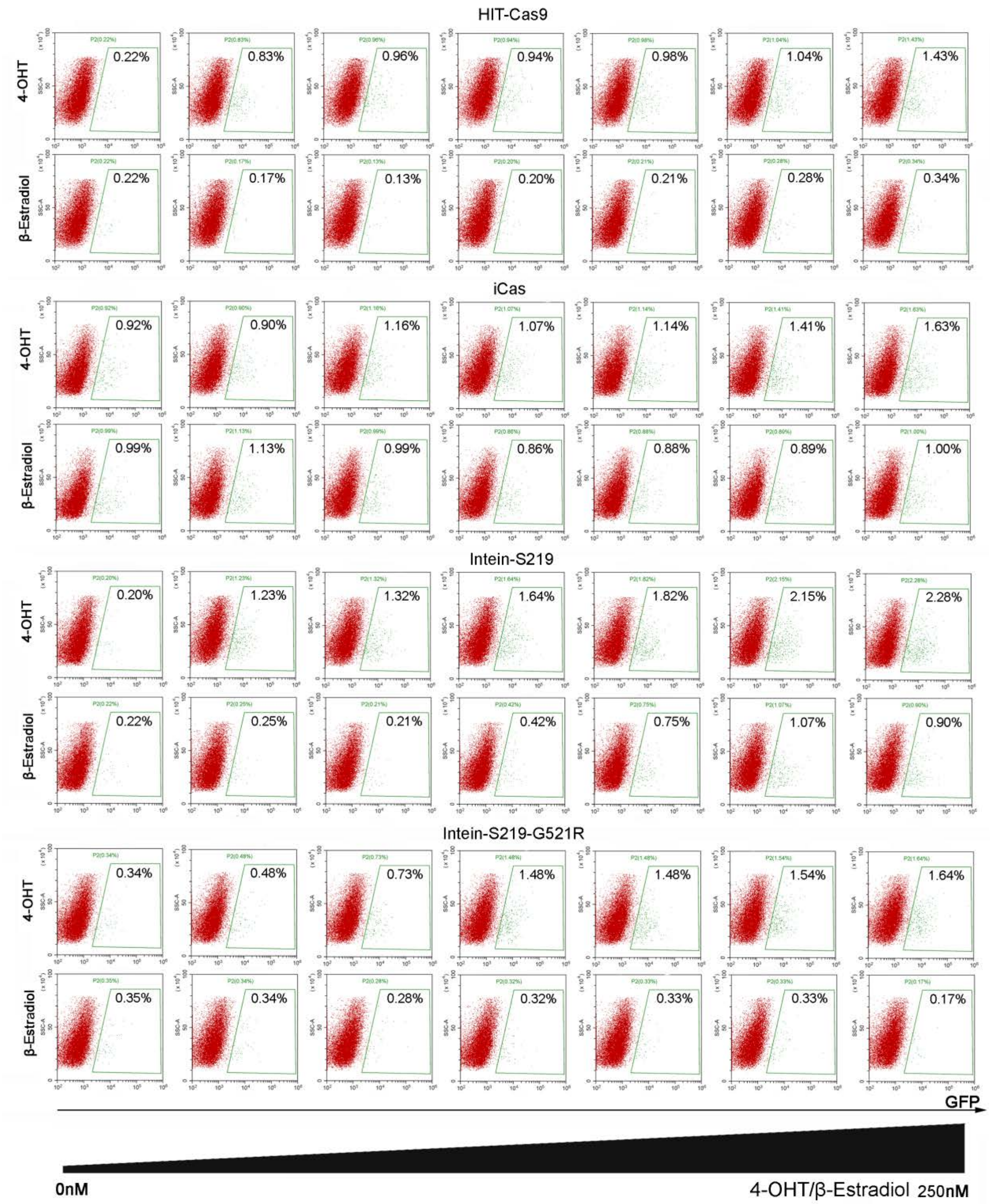

Supplementary Figure 10. Representative flow cytometry plots of dose dependent genome editing by HIT-Cas9 in comparison with other designs using the FCR assay upon treatments of 4-OHT and β-estradiol respectively.

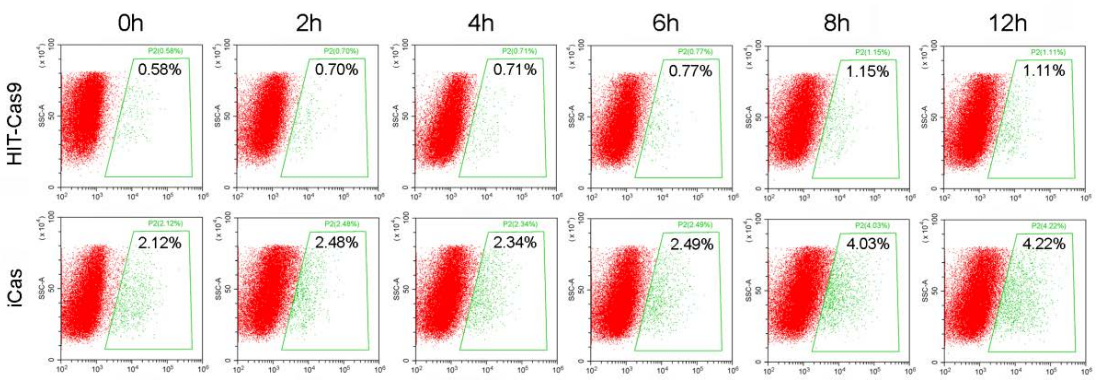

Supplementary Figure 11. Representative flow cytometry plots from the FCR assay to examine the speed of response of HIT-Cas9 and iCas to 4-OHT induction.

Supplementary Table 1.sgRNAs used in this study.

**sgRNAs for gene editing**		
**Target gene or site**		sgRNA target sequence
Human Telomere	sgRNA-1	GTTAGGGTTAGGGTTAGGGTTA
	sgRNA-2	GGGTTAGGGTTAGGGTTA
	sgRNA-3	GGTTAGGGTTAGGGTT
Human Oct4	sgRNA-1	CCCCCGCCGTATGAGTTCTG
	sgRNA-2	TTGGAGACCTCTCAGCCTGA
	sgRNA-3	AGTGAGCTTCGACGGGGTTG
Human CD201		CCTGGTACCACACGTGATAG
**sgRNAfor FCR assay**		
**Target gene**		sgRNA target sequence[21]
BFP		GCTGAAGCACTGCACGCCAT
**sgRNAs for surveyor and TIDE assays**		
**Target gene or site**		sgRNA target sequence[3, 11, 15]
EMX1		GAGTCCGAGCAGAAGAAGAA
EMX1 off-target 1		GAGT**TA**GAGCAGAAGAAGAA
EMX1 off-target 2		GAGTC**TA**AGCAGAAGAAGAA
AAVS1		GGGGCCACTAGGGACAGGAT

Supplementary Table 2. Primers used in surveyor and TIDE assays.

Primer name	Sequence[11, 15]
**EMX1-On target-F**	GCCCCTAACCCTATGTAGCC
**EMX1-On target-R**	GGAGATTGGAGACACGGAGA
**EMX1-Off target 1-F**	TTGAGACATGGGGATAGAATCA
**EMX1-Off target 1-R**	CAGGAATAGCCCTACAAAGGTG
**EMX1-Off target 2-F**	GTTCTGTAAACGCCGTAGCC
**EMX1-Off target 2-R**	GGATGCAGTCTGCCTTTTTG
**AAVS1-On target-F**	CCTATGTCCACTTCAGGACAGC
**AAVS1-On target-R**	CCTGCCAAGCTCTCCCTCCCAG

Supplementary Table 3.sgRNAs used with SaCas9.

**sgRNAs for gene editing**	
**Target gene or site**	sgRNA target sequence
**BFP**	GAAGCACTGCACGCCATGGGT
